# Long Term Pharmacological Perturbation of Autophagy in Mice: Are HCQ Injections a Relevant Choice?

**DOI:** 10.3390/biomedicines8030047

**Published:** 2020-03-01

**Authors:** Jean-Daniel Masson, Benoit Blanchet, Baptiste Periou, François-Jérôme Authier, Baharia Mograbi, Romain K. Gherardi, Guillemette Crépeaux

**Affiliations:** 1 Univ Paris Est Creteil, INSERM, IMRB, F-94010 Creteil, France; jean-daniel.masson@inserm.fr (J.-D.M.); baptiste.periou@inserm.fr (B.P.); francois-jerome.authier@aphp.fr (F.-J.A.); romain.gherardi@aphp.fr (R.K.G.); 2 AP-HP, Hôpital Cochin, Biologie du médicament - Toxicologie, 27 rue du Faubourg Saint-Jacques, 75014 Paris, France; benoit.blanchet@aphp.fr; 3 University Paris Descartes, Faculty of Pharmacy, UMR8038 CNRS, U1268 INSERM, PRES Sorbonne Paris Cité, 75006 Paris, France; 4 Hôpitaux Universitaires Henri Mondor (APHP), Centre Expert de Pathologie Neuromusculaire, 94010 Créteil, France; 5 Université Côte d’Azur, CNRS, INSERM, IRCAN, FHU-OncoAge, Centre Antoine Lacassagne, 06107 Nice France; baharia.mograbi@unice.fr; 6 Ecole Nationale Vétérinaire d’Alfort, IMRB, F-94700 Maisons-Alfort, France

**Keywords:** autophagy, hydroxychloroquine, mice, long term, dose-response

## Abstract

Macroautophagy (hereafter referred to as autophagy) is an evolutionarily conserved catabolic process whose loss-of-function has been linked to a growing list of pathologies. Knockout mouse models of key autophagy genes have been instrumental in the demonstration of the critical functions of autophagy, but they display early lethality, neurotoxicity and unwanted autophagy-independent phenotypes, limiting their applications for in vivo studies. To avoid problems encountered with autophagy-null transgenic mice, we investigated the possibility of disturbing autophagy pharmacologically in the long term. Hydroxychloroquine (HCQ) ip injections were done in juvenile and adult C57bl/6j mice, at range doses adapted from the human malaria prophylactic treatment. The impact on autophagy was assessed by western-blotting, and juvenile neurodevelopment and adult behaviours were evaluated for four months. Quite surprisingly, our results showed that HCQ treatment in conditions used in this study neither impacted autophagy in the long term in several tissues and organs nor altered neurodevelopment, adult behaviour and motor capabilities. Therefore, we recommend for future long-term in vivo studies of autophagy, to use genetic mouse models allowing conditional inhibition of selected *Atg* genes in appropriate lineage cells instead of HCQ treatment, until it could be successfully revisited using higher HCQ doses and/or frequencies with acceptable toxicity.

## 1. Introduction

Autophagy is a detoxification pathway preserving cellular homeostasis in physiological conditions and more importantly under environmental stress by ensuring the turnover of damaged organelles and long-lived proteins [1]. The term “autophagy” was first introduced by Christian De Duve [2] more than five decades ago. Three forms of autophagy are commonly described: microautophagy able to engulf the cytoplasmic cargo by lysosomal membrane invagination [3], chaperone-mediated autophagy, which used specific proteins to internalize cytosolic cargo through the lysosome membrane [4] and macroautophagy (hereafter referred to as autophagy). Autophagy, as protein and organelles major degradative pathway, plays essential roles in cell survival, tissue remodelling and tumour suppression and its dysfunction is crucially involved in a variety of pathologic states [5]. Autophagy is mediated by over 30 autophagy-related (ATG) genes that have been mainly disclosed in *S. cerevisiae* and found well-conserved in mammals. The autophagy process begins with the formation of a double-membrane compartment (“phagophore”) that sequesters cargo from the cytosol. Phagophore expands into a completed vesicle (“autophagosome”) which subsequently fuses with a lysosome (“autolysosome”) allowing degradation of the luminal content by acid hydrolases [6]. Before fusion with a lysosome, the autophagosome can also fuse with an endosome to form an amphisome connecting autophagy with phagocytosis [7]. As a highly dynamic multi-step process, autophagy is hard to measure, and evaluation of autophagy flux remains essential. According to Klionsky’s et al. guidelines [8], several techniques can be used to distinguish increased autophagy induction, impaired autophagosome/lysosome fusion, and the inability to clear autophagosomes, which can all produce the same response. One of the most common methods relies on the quantification of both microtubule-associated protein 1A/1B-light chain 3 (LC3)-I and LC3-II, a specific autophagosome marker, and the degradation of the autophagy substrated SQSTM1/p62 proteins by Western blotting.

Autophagy usually occurs at a basal rate in all cell-type to maintain cellular homeostasis by eliminating misfolded proteins and damaged organelles. However, this process can be induced by stress conditions such as metabolic stress (starvation) or hypoxia and can modulate the oxidative stress or the inflammatory response [9]. A related mechanism is implicated in the digestion of unwanted foreign invading material (“xenophagy”) such as bacteria and viruses. Several ATG and additional genes, ensure the rapid delivery of extracellular cargo to the lysosome through a non-canonical autophagy pathway called LC3-associated phagocytosis (LAP) [10]. Xenobiotics, metals and particles are known to interplay with the autophagy machinery [11], and xeno/autophagy is involved in the biodisposition and toxicity of mineral [12,13] and metallic particles [14,15], which can, in turn, destabilize lysosomes [16,17,18,19,20]. As a result, even at low doses, close to an environmental exposure level, xenobiotics, metals and pesticides are able to suppress autophagy, explaining their adverse toxic and inflammatory effects [21,22,23,24].

Autophagy defects have been involved in a growing list of pathologies, including toxic injury, infections, neurologic, neurodevelopmental, myopathic, autoimmune or inflammatory conditions [25]. Single nucleotide polymorphisms (SNPs) in several autophagy genes have been found in patients with inflammatory bowel disease (IBD) [9,26,27], leading to a reduced expression of ATG16 protein [28,29]. A mouse model expressing the *Atg16L1* T300A variant showed that autophagy deficiency was associated with increased pro-inflammatory cytokine (IL1β) secretion by macrophages [29]. Other genetic models targeting *Atg16L1* or *IRGM* were shown to present higher sensitivity to xenobiotics, like dextran sulphate sodium, than control mice [29,30,31]. Furthermore, it has been proposed that SNPs in autophagy genes may predispose to inflammatory diseases only upon a particular environmental exposure [9]. For example, promising preliminary data have been obtained by DNA screening of 34 genes directly involved in the xeno-autophagy machinery. It is suggesting that the abnormal biopersistance of Aluminium (Al) particle adjuvants observed in the Macrophagic myofasciitis (MMF) lesion may reflect genetically-determined inability of some individuals to efficiently dispose of injected Al adjuvants (European patent 18207583.8-1118, manuscript deposited) [32]. Thus, in addition to the already reported impaired autophagic response to Al oxide particles [16,17,33], it appears that the poor clearance of Al adjuvants in MMF patients could be related to a genetically susceptibility inducing limited autophagy, likely causing longstanding immune stimulation and favouring translocation of the adjuvant-loaded immune cells to distant organs and the brain [34]. Thus, as in IBD in which chronic inflammation has been attributed to lack of autophagic clearance of intracellular microbial particles [35], abnormal sensitivity to vaccine adjuvant particles may well be linked to genetically impaired ability to clear out Al particles, involving autophagy deficiency [32].

Altogether, those observations highly suggest that impaired autophagy and/or altered Al particle detoxification may be involved in Al-particle induced neurotoxicity and MMF pathogenesis. This prompted us to develop an in vivo model to further study the consequences of autophagy inhibition on toxicity and MMF pathogenesis. The most common way to model autophagy-deficiency conditions in vivo is to use genetically modified mice with deleted *ATG* genes, but this approach presents some disadvantages. Among all genes controlling autophagy, 14 have been knocked out in mice. Moreover, due to the multiple and crucial functions of autophagy at different developmental stages, *Atg* null mice are often nearly unusable: some KO mice die *in utero*, other die a few days after birth prohibiting long term studies, and the viable animals may show dysfunctional phenotypes interfering with planned analyses [36]. For example, *Atg5* KO and *Atg7* KO are lethal at very early stages of life, and, when KO was restricted to myeloid lineage cells, different unexpected phenotypes may ensue: *Atg7* KO in myeloid lineage cells impairs synaptic pruning and causes social behavioural defects in mice pups [37] whereas *Atg5* KO in myeloid lineage cells is associated with abnormal intracellular proteins accumulation in neurons accompanied by neurodegeneration and progressive deficit of motor function [38].

To circumvent genetic model limitations, we searched for a pharmacological method to globally disturb autophagy after birth, in both juvenile and adult mice. Hydroxychloroquine (HCQ), and chloroquine (CQ), are drugs used to treat malaria and have anti-inflammatory properties that have long been recognized and used to treat rheumatoid arthritis and systemic lupus erythematosus [39]. HCQ differs from CQ only by a hydroxyl group, but this difference allows it to be three time less toxic than CQ [40,41]. It is a fully water-soluble diprotic weak base, highly attracted by acidic compartments (lysosomotropic agent). It can cross the lysosome’s membrane under unprotonated form. Once protonated, HCQ stays trapped in lysosomes, where it induces pH increase by proton capturing [42]. Interference with lysosomal acidification leads to default in phagolysosomal fusion [43]. HCQ kinetics are a bit complex with a quick plasma concentration peak 4 to 12 h after injection, followed by an exponential decrease with a half-life of 74 h to 50 days, depending on initial dosage [42,44]. HCQ and CQ are currently and successfully used to induce short time perturbations of the autophagy pathway through intraperitoneal (ip) injections in mice and rats [45,46,47,48,49,50]. Doses used for mice treatment range from 10 to 80 mg/kg with the effect observed from some hours to some days after the last injection, with a maximum treatment duration of two weeks in the literature [50,51,52,53,54,55]. Moreover, CQ and HCQ are recommended as autophagy inhibitors for the autophagy flux measurement in vitro [8,56,57]. However, doses used and schedule applied should be carefully selected because CQ and to a lesser extent, HCQ, are toxic compounds able to produce vacuolar myopathy [58,59], retinopathy [60], cardiomyopathy and neuromyopathy [42]. As a lysosomotropic drug such as CQ, HCQ allows LC3-II and SQSTM1/p62 to accumulate as a probable result of lysosome deprotonation and/or inhibition of the fusion between lysosome and autophagosome, and the subsequent impairment of autolysosomal degradation [50].

In the present study, we aimed to develop a murine model of long-term systemic autophagy dysfunction, by the way of repeated injections of HCQ in both juvenile and adult animals, while ensuring a limited impact on development, behaviour and motricity. This model could allow to perform long-term toxicokinetic studies and to understand the role of autophagy in toxicity and kinetic of immune-stimulating particles.

## 2. Materials and Methods

In this study, two experiments were carried out in parallel: the first one included juvenile mice treated from birth to their fourth month and the second one included adult. Juvenile mice were tested for neurobehavioural development between post-natal day (PND) 5 to 25 and were then evaluated with a battery of behavioural or physical tests during one week before PND73 and PND136. Adult mice were evaluated with the same array of tests than juvenile mice with the same delay elapsed from the beginning of treatment and behavioural evaluation.

### 2.1. Animals

#### 2.1.1. Juvenile Experiment

Twenty-eight females (7 weeks) and 14 males (8 months) C57BL/6JRj mice were purchased from Janvier Labs (France). Upon arrival, females were housed 5 per cage, and males were housed lonely in a regulated environment (temperature 22 ± 2 °C; humidity 55 ± 10%) under a 12 h light cycle (lights on from 7:00 a.m. to 7:00 p.m.). Animals were given free access to food (Granovit AG, Kaiseraugst, Switzerland) and water. After one week for acclimatization, females were mated with breeding males (two females with one male) and were examined the following evening by vaginal smear to assess successful mating. When successful, females were removed from the cage and were housed individually. At birth (day of birth = post-natal day PND0), litters were reduced to 6 pups if necessary to standardize the litter size and to prevent any other litter effects on pup development. All pups were tested for developmental behaviour from PND5 to PND25.

Males and females were separated after weaning (PND28). Adult behavioural tests were performed during a week from PND73 and PND136. At PND11, 26, 73 and 136, seven animals per gender per treatment were sacrificed, and samples removed for Western-blotting analyses and analytical protocol (see Section 2.3).

All the animal experiments were performed following the rules provided by the European Union (Directive 2010/63/EU) and were approved by the institutional ethics committee of the National Veterinary school of Maisons-Alfort (authorization number APAFIS#13038-2018011509088600, 23 August 2018).

#### 2.1.2. Adult Experiment

A total of 60 female and 60 male (8 weeks) C57BL/6JRj mice were purchased from Janvier Labs (France). Upon arrival, animals were housed 5 per cage in a regulated environment (temperature 22 ± 2 °C; humidity 55 ± 10 %) under a 12 h light cycle (lights on from 7:00 a.m. to 7:00 p.m.). Animals were given free access to food (Granovit AG, Kaiseraugst, Switzerland) and water. After one week for acclimatization, animals were randomly assigned to one of the experimental groups. Day 1 (D1) of this adult experiment is defined as the first injection day. Animals were sacrificed at three different endpoints: one at the beginning of the experiments, one two months later, and one four months and a half (exact days 3, 73 and 136 after the first injection). Samples removed for Western-blotting analyses and analytical protocol (see Section 2.3). Animals were tested for behaviour before the two last endpoints (73 or 136 days after the first HCQ injection).

All experiments were performed following the rules provided by the European Union (Directive 2010/63/EU) and were approved and supervised by the institutional ethics committee of the National Veterinary school of Maisons-Alfort (authorization number APAFIS#9767-2017042116187889, 30 November 2017 and APAFIS#11633-2017100309523784, 6 April 2018).

### 2.2. Doses and Protocol of Exposure

#### 2.2.1. Juvenile Experiment

Five experimental groups were formed, each including a minimum of 5 animals/sex/endpoint. In each litter, animals were randomly assigned to one of the HCQ-dose group. The five groups were exposed to 0; 15; 30; 50 or 70 mg of hydroxychloroquine (HCQ) per kg through ip injections, at PND6, 10, 14, 21 then once a week until this end of the experiment. From PND6 to PND21 mice received a common injection volume of 25 µL, from PND22 to PND35 the injected volume was 100 µL and then 200 µL for adult mice. Doses used were calculated from the CDC human recommendation of 310 mg per week. A 75 kg man taking this medicine receives around 4 mg of HCQ per kilogram of body weight. We used an allometry calculation based on body surface area that reflects the metabolic rate to determine the human equivalent dose per kg to extrapolate human to mouse dosage. This x12.3 allometric conversion factor from human to mouse [61] is easy to apply. The conversion resulted in 50 mg HCQ/kg mouse body weight for one human dose. This basis dose was subsequently adjusted with a stronger one (70 mg/kg) if mice were resistant to HCQ action and two small doses (15 and 30 mg/kg) to prevent animal damages if mice were responsive to treatment especially the dose 15 mg/kg for juvenile which were not fully developed and probably more sensitive to pharmacological therapies. HCQ (Plaquenil^®^, Sanofi-Aventis, Gentilly, France) was prepared to mimic the human prophylactic treatment against malaria which can be maintained in the long term. Some components of the pills are probably able to modulate the autophagy process (e.g., excipients), therefore we have chosen to work with the whole human treatment and not the only active principle. Plaquenil^®^ pills were grinded and dissolute in physiological serum to obtain the desired concentration of HCQ by vortex homogenizer. HCQ solutions were prepared extemporaneously. Concentrations of HCQ were calculated according to the mean weight of each group to fix a common injected volume for all animals.

#### 2.2.2. Adult Experiment

After the acclimatization period, four experimental groups were formed, each including 5 animals/sex/endpoint. The four groups were exposed to 0; 30; 50 or 70 mg of hydroxychloroquine (HCQ) per kg through ip injections with a common volume of 200 µL, at D1, 2, 6, 10 then once a week until this end of the experiment. The distribution of the animals within the groups of exposure is presented in Table 1. HCQ was prepared from Plaquenil^®^, as explained above.

### 2.3. Removed Sample Analyses

#### 2.3.1. Tissue Preparation

A whole litter was sacrificed by decapitation at PND11 or cervical dislocation at PND26, 73 and 136 to reach a total number of 5 animals/sex/group and samples removed for Western-blotting analyses. The following samples were removed intact, flash-frozen in liquid nitrogen, and stored at −80 °C: anterior tibialis anterior muscle (TA), popliteal and inguinal lymph nodes, liver, spleen and brain.

Five animals/sex/group was sacrificed by cervical dislocation at D3, D73 and D136, and samples removed for western-blotting analyses and analytical protocol. The following samples were removed intact, flash-frozen in liquid nitrogen, and stored at −80 °C: anterior TA, popliteal and inguinal draining lymph nodes, liver, spleen, brain and blood.

#### 2.3.2. Analytical Dosage of HCQ

After sampling, blood was let at room temperature (RT) 30 min to coagulate, and then it was centrifuged 10 min at 1000× *g* and RT. After the centrifugation, the supernatant (serum) was carefully removed and store at −80 °C. The method used for HCQ assay in serum was adapted from a previously published method [62]. The intraday and interday precision ranged from 4.3% to 10.3%. The lower limit of quantification in serum was 25 ng/mL.

#### 2.3.3. Western Blot of Autophagy Proteins

For both juvenile and adult experiments, removed organs from 5 animals/sex/group were homogenized in lysis buffer (RIPA) supplemented with protease phosphatase inhibitor (Fisher Scientific, A32959, Illkirch, France) using precellys homogenizer (Bertin Instruments, Montigny-le-Bretonneux, France) and clarified by centrifugation. Protein quantifications were performed by Pierce™ BCA Protein Assay Kit (Fisher Scientific, 23225, Illkirch, France) and an equal protein mass of 10 µg in 10 µL was subjected to NuPAGE™ 10% Bis-Tris Midi Protein Gels (Invitrogen™, WG1203BOX, Illkirch, France) in Xcell4 Surelock tank (Life Technology SAS, WR0100, Illkirch, France) using NuPAGE™ MES SDS Running Buffer (20X) (Invitrogen™, NP000202, Illkirch, France). Protein transfer to polyvinylidene difluoride (PVDF) membrane was performed using iBlot2 Dry Blotting System (Fisher Scientific, IB21001, Illkirch, France) and iBlot™ 2 Transfer Stacks (Invitrogen™, IB24001, Illkirch, France). Membranes were blocked in cold water fish skin gelatin solution 2.5%, then probed with rabbit anti-LC3B (1:1000; Life Technology, PA1-46286, Courtaboeuf, France) or rabbit anti-SQSTM1 (1:3000; Life Technology, PA5-20839, Courtaboeuf, France) overnight at 4 °C. Membranes were then washed and exposed (60 min) to HRP-conjugated goat anti-rabbit secondary antibody (1:5000; Santa Cruz, sc-2054, Heidelberg, Germany) or mouse anti-β-actin HRP-conjugated (1:10,000; Santa Cruz, sc-47 778 HRP, Heidelberg, Germany). Proteins were visualized by an enhanced chemiluminescence assay kit (SuperSignal™ West Femto; Fisher scientific, 34096, Illkirch, France) using a c600 scanner (Azure Biosystems, Inc., Dublin, OH, USA) and signals were quantified using ImageJ software (V1.52s, National Institutes of Health, Bethesda, MD, USA). To perform level expression comparison, the quantification of each protein was divided by the β-actin quantification, which was the normalizing protein used in this study.

### 2.4. Behavioural Testing

#### 2.4.1. Neurobehavioural Development in Juvenile Experiment

In addition to behavioural testing, daily inspections were made to assess the effects of HCQ exposure on pup body weight, hearing sensitivity (PND12-13) and eye-opening (PND15-16). All pups were subjected to a whole testing battery to evaluate motor coordination and vestibular function in righting reflex test (PND5), grasping reflex (PND6), ability to perceive cliff and gravity in cliff avoidance (PND7) and negative geotaxis tests (PND8), muscular strength in forelimb grip strength test (PND10), locomotor coordination in water escape climbing (PND23) and locomotor activity in open field (PND25). All these tests, being standardized to the normal maturation of the central nervous system in rodents [63,64,65,66,67,68,69], were performed in a separated room, under a red light, between 9 a.m. and 13 p.m. The testing order was randomized between animals from the five groups of exposure to avoid circadian variation. A total number of 210 pups including both males and females, were tested up to PND25. The distribution of the animals within the groups of exposure is presented in Table 1.

For more details on neurobehavioural development in juvenile see supplementary materials.

#### 2.4.2. Maternal Behaviour in the Juvenile Experiment

Maternal behaviour was also assessed to avoid any influence of maternal care on pup development. It also confirms that the injections of HCQ in young mice do not impact their mother’s behaviour towards them. Nest building was conducted at PND3, 6 and 8, and the retrieving test was performed at PND4, 7 and 10. These two tests allowed evaluating the level of care given by the mother to pups. For more details, see supplementary materials.

#### 2.4.3. Mature Behavioural Testing

For both juvenile and adult experiments, four behavioural or physical tests were performed the week before D73 or before D136 for adult experiment and before PND73 or PND136 for the juvenile experiment. Tests were chosen to assess locomotor activity in the open-field [70], muscular strength in the wire mesh hang [71], and grip strength test [72] and locomotor coordination in the rotarod test [73]. These tests were shared on four days: the first day was dedicated to the first rotarod training, the second day to grip strength test followed by wire-mesh hang test, then the second rotarod training, the third day to accelerating rotarod test and the fourth to the open field test. For more details, see supplementary materials.

### 2.5. Statistical Analysis

To determine the use of parametric vs. nonparametric tests, data were analysed by the Shapiro–Wilk test assessing distribution normality.

- Data from behavioural developmental tests were analysed using a non-parametric Kruskal–Wallis test followed by a Mann–Whitney procedure modified for multiple comparisons when appropriate. Pearson Chi-square procedure was used to analyse the number of successful animals. All males and females were tested together up to PND25 then gender was used as a variable for analysis.
- Pup weight was analysed using a one-way analysis of variance (one-way ANOVA). Post hoc comparisons have been performed using the Dunnett’s test when ANOVA was significant.
- Data from adult behavioural testing in both experiments were analysed using a one-way analysis of variance (one-way ANOVA). Post hoc comparisons have been performed using the Dunnett’s test when ANOVA was significant.
- Western-blot data and analytical HCQ dosages were analysed using a non-parametric Kruskal–Wallis test followed by a Mann–Whitney procedure modified for multiple comparisons when appropriate, due to the low number of animals in each group. For statistical comparison reasons, each gender was analysed separately because they were generated with two successive Western blots and multiple comparisons were exclusively performed for treated groups compared to controls.

Significance was set at *p* < 0.05. All statistical analyses were carried out using SPSS 25 software (SPSS INC., Chicago, IL, USA).

## 3. Results

### 3.1. HCQ Assay in Plasma

This dosage was conducted to monitor serum HCQ concentration and to check whether blood accumulation of HCQ remains low during the four months of treatment in adults. HCQ was not detected for any control animal and was found in the range of 0 to 415 ng/mL in treated animals.

**Figure 1:**
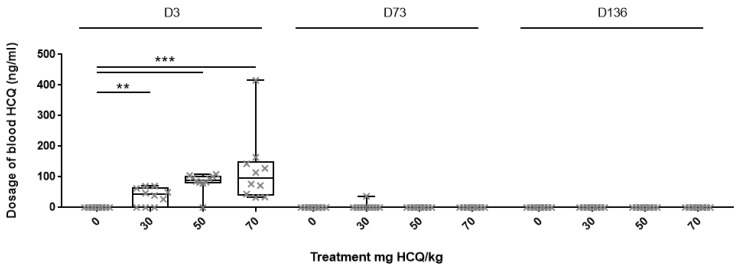
Serum hydroxychloroquine (HCQ) levels in C57BL/6JRj mice treated with intraperitoneal injection of HCQ. Each cross represents one animal’s dosage. Boxplot expressed median and quartile. Dosages were analysed by the Kruskal–Wallis test. Mann–Whitney was used for multiple comparisons. ** *p* < 0.01; *** *p* < 0.001.

At D3, HCQ was detected in 7 of 11 animals (63%) from group 30 mg/kg, in 9 of 10 animals (90%) from group 50 mg/kg, and in 10 of 10 animals (100%) from group 70 mg/kg. The median serum HCQ concentration was 39 ng/mL, 90 ng/mL and 96 ng/mL in 30 mg/kg, 50 mg/kg and 70 mg/kg group, respectively (*p* < 0.001) (Figure 1). Mann–Whitney test modified for multiple comparisons confirmed higher serum concentration of HCQ in mice treated with 30 mg/kg (*p* < 0.01), 50 and 70 mg/kg (*p* < 0.001) compared to untreated at D3 (Figure 1). At D73 and D136, all serum HCQ concentrations were below the lower limit of quantification (<25 ng/mL) except one from group 30 mg/kg at D73 (37 ng /mL, not considered for statistical analysis).

**Figure 2:**
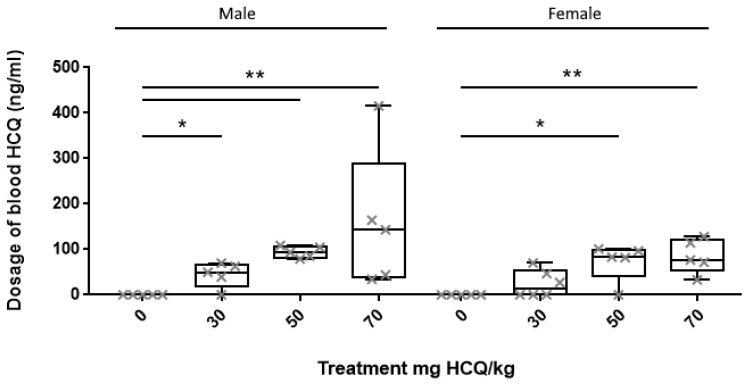
Dosage of blood HCQ expressed by gender at D3. Each cross represents one animal’s dosage. Boxplot expressed median and quartile. Dosages were analysed by the Kruskal–Wallis test. Mann–Whitney was used for multiple comparisons. * *p* < 0.05; ** *p* < 0.01.

When serum HCQ concentration was analysed per gender, male median serum HCQ concentration was 50 ng/mL, 94 ng/mL and 143 ng/mL in 30 mg/kg, 50 mg/kg and 70 mg/kg group, respectively (*p* < 0.01) and female median serum HCQ concentration was 14 ng/mL, 83 ng/mL and 77 ng/mL in 30 mg/kg, 50 mg/kg and 70 mg/kg group, respectively (*p* < 0.01) (Figure 2). Mann–Whitney test modified for multiple comparisons confirmed higher male serum HCQ concentrations in group 30 mg/kg (*p* < 0.05), 50 and 70 mg/kg (*p* < 0.01) as well as higher female serum concentration in group 50 mg/kg (*p* < 0.05) and 70 mg/kg (*p* < 0.01), compared to controls (Figure 2). There was no difference of HCQ serum concentration between male and female for any treatment.

### 3.2. Western Blot of Autophagy Proteins

#### 3.2.1. Juvenile Experiments

Results for protein quantification comparisons are summarized in Table 2.

Compared to controls, some proteins were accumulated or depleted in treated animals without apparent rule or organization (Table 2).

PND11:

Male skeletal muscles, spleen and brain were impacted by the pharmacological treatment with a majority of protein decrease. LC3-II was significantly depleted in skeletal muscle for groups 30, 50 and 70 mg/kg and in the brains for all HCQ groups. SQSTM1/p62 was more present in skeletal muscle for group 50 mg/kg and less present in the spleen for group 30 mg/kg than controls.

Female lymph nodes showed a statistically significant LC3-II accumulation for group 30 mg/kg and a SQSTM1/p62 increase for groups 15 and 30 mg/kg.

PND26:

The pharmacological treatment statistically modulated male skeletal muscles, spleen and brain protein amount. LC3-II was accumulated in skeletal muscle for group 30 mg/kg and in the brain for groups 15, 30 and 50 mg/kg. SQSTM1/p62 was found in higher quantity in the spleen for group 15 mg/kg and in the brain for groups 15, 50 and 70 mg/kg but in lower quantity in the spleen for group 70 mg/kg.

Female skeletal muscle, draining lymph nodes, spleen and brain expressed statistically proteins amount modulation. LC3-II was accumulated in the brain for group 50 mg/kg and SQSTM1/p62 amount was increased in skeletal muscle for groups 15 and 30 mg/kg and decreased in the spleen for groups 30, 50 and 70 mg/kg.

PND73:

Male organs did not present protein amount modification for any groups of treatment.

Female lymph nodes, spleen and liver were impacted by the pharmacological treatment. LC3-II amount was increased in lymph nodes for group 30 mg/kg and decreased in the spleen for groups 15 and 30 mg/kg. SQSTM1/p62 was accumulated in lymph nodes for groups 30 and 70 mg/kg and reduced in the spleen for groups 15 and 30 mg/kg.

PND136:

Male lymph nodes showed a statistically significant LC3-II accumulation for groups 30 and 70 mg/kg.

Female skeletal muscle, spleen, liver and brain protein quantities were statistically modulated by the treatment. LC3-II was significantly depleted in skeletal muscle for group 50 mg/kg and in the brain for groups 50 and 70 mg/kg but accumulated in the liver for all groups. SQSTM1/p62 was found in lower quantity in skeletal muscle for group 50 mg/kg and in higher quantity in the spleen for group 30 mg/kg.

**Figure 3:**
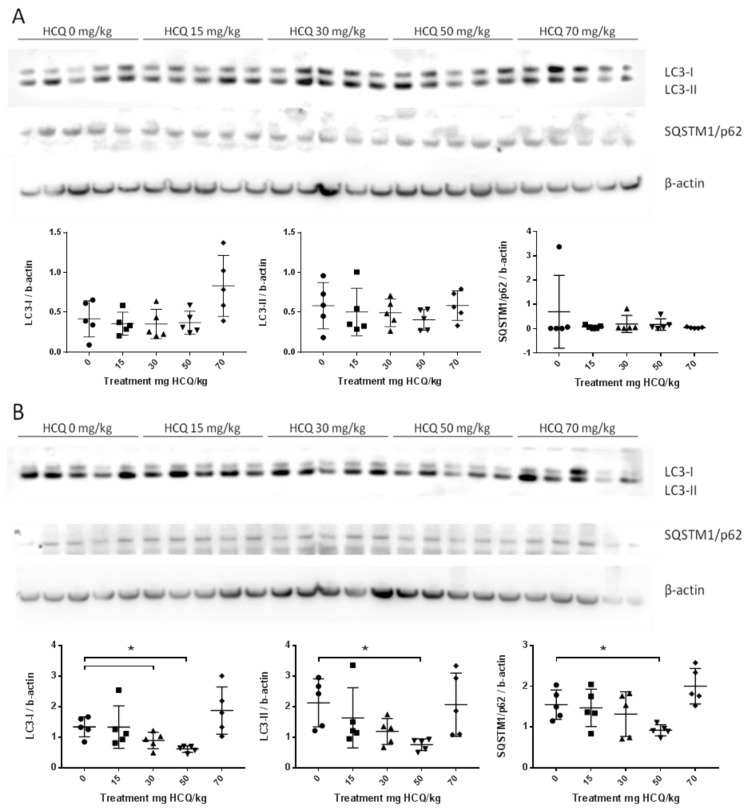
Representative western blots of LC3 and SQSTM1/p62 proteins in the anterior tibialis muscle of juvenile mice sacrificed at post-natal day (PND)136 showing the inter-individual and inter-group variation of protein expression: (**A**) Male data *n* = 5/group; (**B**) Female data *n* = 5/group. Standardized protein levels were analysed by the Kruskal–Wallis test. Mann–Whitney was used for multiple comparisons; * *p* < 0.05.

Finally, no organ appeared to be selectively impacted by the HCQ treatment at any endpoint. There was no accumulation of LC3-II and SQSTM1/p62 except in lymph nodes of females treated with 30 mg HCQ/kg at PND11 and PND73, but these effects did not appear to be stable in time, as confirmed by results at PND26 and PND136 (Table 2). Moreover, an additional analysis using LC3-II/LC3-I ratio was conducted to confirm the first result. The LC3 ratio was not modified in any organs, but in males’ brain at PND26 for the group 15 mg/kg, at the same time of SQSTM1/p62 accumulation (data not shown). As distant organs from the injection site, TA muscle, did not present the accumulation of protein LC3-II or SQSTM1/p62 for both males and females (Figure 3). 

#### 3.2.2. Adult Experiments

Results for protein quantification comparisons are summarized in Table 3.

As in juvenile experiments, some proteins were significantly accumulated or depleted compared to controls, without any apparent rule or organization (Table 3)

D3:

Male lymph nodes and liver were impacted by the pharmacological treatment. LC3-II was statistically downregulated in the liver for group 70 mg/kg and the SQSTM1/p62 amount was lower in the liver for group groups 50 and 70 mg/kg.

Female lymph nodes and brain protein quantities were statistically modulated by the treatment. LC3-II was accumulated in lymph nodes for groups 50 and 70 mg/kg. SQSTM1/p62 was statistically higher in lymph nodes for group 70 mg/kg and lower in the brain for group 30 mg/kg.

D73:

Male skeletal muscle, spleen and liver protein quantities were statistically impacted by the treatment. SQSTM1/p62 was depleted in skeletal muscle for group 70 mg/kg, in the spleen for group 50 mg/kg and in the liver for groups 50 and 70 mg/kg.

Female spleen and brain were impacted by the pharmacological treatment. LC3-II amount was lower in the brain for group 50 mg/kg, and SQSTM1/p62 quantity was lower in the spleen for group 70 mg/kg.

D136:

All male organs expressed proteins amount modulation. LC3-II was more concentrated in lymph nodes for groups 30 and 50 mg/kg and less concentrated in the spleen for group 50 mg/kg, in the liver for group 70 mg/kg and in the brain for groups 30 and 70 mg/kg. SQSTM1/p62 was depleted in skeletal muscle for group 50 mg/kg, in lymph nodes for group 30 mg/kg, in the spleen for all groups and in the liver for group 70 mg/kg.

Female lymph nodes, spleen and liver presented an affected amount of proteins. LC3-II was accumulated in the liver for all groups and SQSTM1/p62 amount was significantly lower in the spleen for group 70 mg/kg but higher in the liver for all groups.

**Figure 4:**
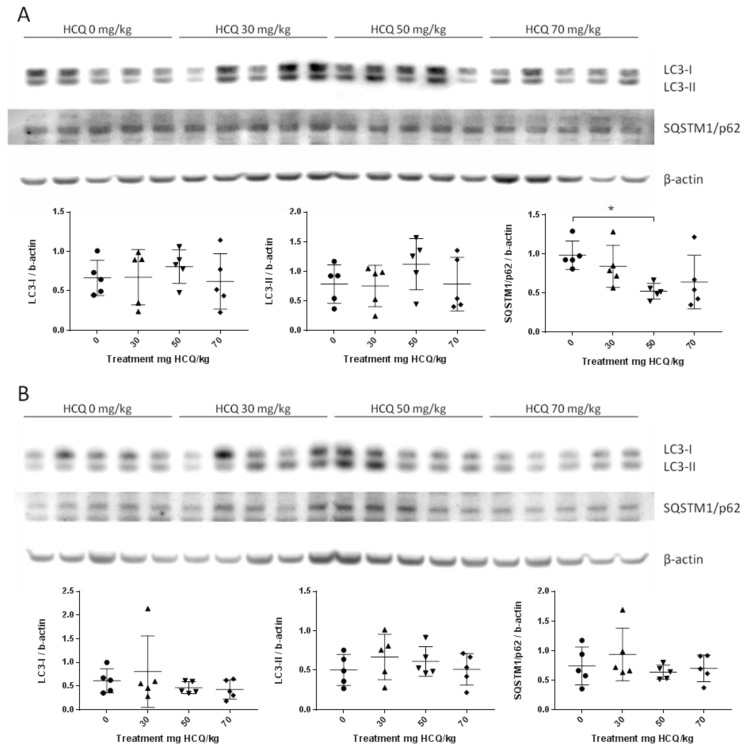
Representative Western blot of LC3 and SQSTM1/p62 proteins in the anterior tibialis muscle of adult mice sacrificed at PND136 showing the inter-individual and inter-group variation of protein expression: (**A**) Male data *n* = 5/group; (**B**) Female data *n* = 5/group. Standardized protein levels were analysed by Kruskal–Wallis test. Mann–Whitney was used for multiple comparisons; * *p* < 0.05.

The expected accumulation of LC3-II and SQSTM1/p62 was only observed in the liver of females at D136 (Table 3). Moreover, the additional analysis using LC3-II/LC3-I ratio was conducted to confirm the first result. The LC3 ratio was not modified in any organs at the same time of SQSTM1/p62 accumulation (Data not shown). As distant organ from the injection site, TA muscle, did not show accumulation of protein LC3-II or SQSTM1/p62 for both males and females (Figure 4). 

### 3.3. Behavioural Testing

#### 3.3.1. Juvenile Experiments

No effect of HCQ exposure on pup growth was observed since the weight of pups was not statistically different across all groups from PND3 to PND25 (Table S1). Similarly, neurobehavioural and motor tests yielded no salient changes in righting reflex, grasping reflex, cliff avoidance, negative geotaxis, forelimb grip strength, hearing pups, eye opening pups, water escape pole climbing and open field test (see supplementary materials). Results for developmental tests are summarized in Table S2.

Furthermore, the nest building test as well as the retrieving test performed to assess maternal behaviour over the first 10 days of post-natal life did not highlight an HCQ-treatment effect on maternal care (data not shown).

Mature mice body weight was not statistically different between treated and untreated mice from PND26 to PND136 (Table S3). Analyses showed significant differences between males and females. No differences were reported between groups of treatment except at PND95 but post-hoc analysis refutes difference between treated groups and controls. There was no significant difference between treated and untreated animals in open field, wire-mesh hang test, grip strength test and accelerating rotarod (see supplementary materials). Results for mature behavioural tests are summarized in Table S4 for results at PND73 and in Table S5 for results at PND136.

#### 3.3.2. Adult Experiments

No effect of HCQ exposure on adult mice body weight was observed since the body weight was statistically equivalent between treated and untreated mice from D1 to D136. As expected, analyses showed significant differences between males and females (Table S6). Such as in mature behavioural testing, no significant change was highlighted by statistical analysis between treated and control mice in open field, wire-mesh hang test, grip strength test, and accelerating rotarod (see supplementary materials). Results for adult behavioural tests are summarized in Table S7 for results at D73 and in Table S8 for results at D136.

## 4. Discussion

In order to determine if HCQ treatment can down-regulate autophagy on the long term and mimic a functional alteration of autophagy, we performed ip injections of HCQ in adult and, presumably more sensitive, juvenile mice.

The monitoring of HCQ serum levels showed a dose-dependent increase at D3, and this was not found at later time points. Notably, blood collection for HCQ assay in serum at D3 was done 24 h after the last HCQ ip injection, while, according to our injection schedule, blood samples assayed at D73 and D136 were collected one week after the last injection. Additionally, the red blood cell to plasma partition coefficient is high for HCQ in mice [74], suggesting that whole blood concentrations exceed plasma concentrations. Taken together, these elements could explain why most of serum HCQ levels were below the lower of quantification (<25 ng/mL) at these time points. In addition, HCQ is known to have a long steady-state half-life about 22 days and up to 50 days [44]. It also has complex kinetics with a quick plasma peak concentration 4 to 12 h after a single dose and steady-state is reached in plasma after 4 to 6 weeks of daily dosing [42]. Our pharmacokinetic data suggest that a weekly injection of HCQ would not be sufficient to reach pharmacokinetic steady-state in the mouse.

Nevertheless, tissue accumulation of HCQ is known to occur, as documented in skeletal muscle [59], and it remained plausible that biological effects on autophagy could be detected at this level. HCQ action interferes with lysosomal acidification by deprotonation and may leads to default in phagolysosomal fusion such as its homologous, the CQ, do [50]. The lack of fusion between lysosome and autophagosome or the lack of acidic activity in lysosome impedes the degradation of LC3 and SQSTM1/p62 proteins by lysosomal acidic hydrolases and so are accumulated in cells. Western blot of LC3 and SQSTM1/p62 proteins was therefore used to evaluate autophagy disturbance according to Klionsky’s et al. guidelines [8] in order to follow the efficacy of treatment [56,75,76]. Despite the plausible method’s limitations [56], a second evaluation was conducted using LC3-II/LC3-I ratio to check the autophagy flux. In fact, their expected protein accumulation was very rarely observed in the different tested tissues and organs (Table 2 and Table 3) and no correlation with the administered dose was found.

Finally, neurodevelopmental tests [63,64,65,66,67] and behavioural tests evaluating activity, anxiety, locomotor coordination and strength were unable to document early or subtle signs of brain dysfunction. Juvenile mice neurodevelopment was very similar in treated and untreated animals, and these mice once reaching adulthood or mice treated directly at the adult stage exhibited normal behaviour and motricity. The only differences were based on gender only, with proactive and more anxious females compared to males, as previously documented [77,78].

In published studies, HCQ effects can generally be observed rapidly, but it seems that doses and injection schedules we used were unable to stably disturb autophagy in the long term. Of note, we used HCQ at doses similar to what has been reported in the literature, ranging from 10 to 80 mg/kg, that are relevant to human curative treatment but with a lower frequency of injection. Indeed, short term rodent studies generally use a daily injection of pharmacological treatment [45,48,50,79,80,81,82,83] but this daily schedule is inappropriate for long term studies. Therefore, instead of basing the injection schedule on curative HCQ treatment, we based the schedule on the prophylactic HCQ treatment in humans which recommends weekly injections of the pharmacological agent [84].

However, it appears that at the conventional HCQ doses we used the quick dynamic of HCQ would forbid a reduction of injection frequency. Higher doses of treatment could have exposed to high toxicity even using HCQ, which is less toxic than CQ [40,41]. Indeed, detrimental effects for skeletal muscle have been reported after two months of intraperitoneal injection of 10 mg/kg CQ five times per week [58] or after two months of daily subcutaneous injection of 15 mg/kg CQ [59]. HCQ administered at high concentration and too high frequency, induce retinopathy, cardiomyopathy, neuromyopathy and myopathy [42,60]. Searching for a compromise between ethical acceptance in terms of injections number and toxicity of the drug was finally unsuccessful. It is possible that HCQ toxicity was sufficiently low to allow more important doses without causing injury [40,49] but the repeated ip administration on a long time in large cohorts of mice could cause stress to the animals, inducing aggressive behaviours and modulation of immune responses, thus interfering with data read-out.

HCQ is usually administered orally. Though ip injection has been very commonly used [45,46,48] this might not be the optimal mode of delivery. Admittedly, however, ip injections offer several advantages, including precise knowledge of the delivered dose, thus avoiding uncertainties linked to voluntarily liquid intake, rapid administration adapted to studies of large animal cohorts and the limited stress they induce compared to oral gavage [85].

The mouse model we used can also be questioned. Mice might be less sensitive to HCQ than humans rendering the human-based doses ineffective even after the correction we did to take into account the mouse metabolic status. Indeed, Haspel et al. [46] showed that a high dose of CQ, i.e., single ip injection of 60 mg/kg, was required to obtain a significant increase in LC3b in the liver four hours after injection in mouse. Moreover, we used the inbred mouse strain C57bl/6j as commonly done in immunological and autophagy studies [45,50]. However, this mouse strain exhibits a five-exon deletion of nicotinamide nucleotide transhydrogenase (*Nnt*) on chromosome 13, leading to a complete absence of NNT protein [86]. NNT is an inner mitochondrial membrane redox-driven proton pump involved in regenerating NADPH [87]. Moreover, NNT plays a significant role in the modulation of an immune response and C57bl/6j macrophages could exhibit more reactive oxygen species (ROS) and a stronger inflammatory response to pathogens [88] and probably to non-microbial particles, like Plaquenil^®^ excipients. Increasing ROS production could be followed by a lysosomal disruption [89] which liberates several components in the cytosol, increasing the stress and triggering autophagy [12]. Our inability to document autophagy inhibition under HCQ treatment could reflect an inherent condition of C57bl/6j mice to offset the drug action in the long term.

HCQ is a passive lysosomotrophic drug, but proton pumps are still functional and can compensate pH increase. Indeed, H^+^ ions are trapped into the acidic vacuole by the action of the ATP-dependent pumps, attracting HCQ by the pH gradient generated, leading to an accumulation of HCQ in lysosome, which causes an elevation of pH due to H^+^ ions trapped by HCQ [90]. pH must be maintained around 5.0 to assure the optimal activity of hydrolases [91] and proton pumps might try to compensate the pH elevation produced by HCQ presence until HCQ saturation and pH restoration. This proactive loop could lead to a rapid overload of HCQ and habituation to HCQ presence by proton pump reinforcement.

## 5. Conclusions

Despite their potential disadvantages, genetic models probably remain the most appropriate approach to mimic long-term autophagy deficiency. Homologous recombination and Crispr methods allow producing viable mice with specific tissue genetic KO like *Atg7* KO and *Atg5* KO in myeloid lineage [37,38]. Some other models like *Map1lc3*b KO [92], *Ulk1* KO [93] or *Irgm*1 KO [94] are available and seem free of obvious abnormality [36]. Therefore, we recommend for future long-term in vivo studies of autophagy, to use genetic mouse models allowing conditional inhibition of selected *Atg* genes in appropriate lineage cells instead of HCQ treatment, until it could be successfully revisited using higher HCQ doses and/or frequencies with acceptable toxicity.

## Figures and Tables

**Table 1:** Distribution of animals.

A	Juvenile Experiment										Adult Experiment							
	Group (mg HCQ/kg)										Group (mg HCQ/kg)							
	0		15		30		50		70		0		30		50		70	
	Males	Females	Males	Females	Males	Females	Males	Females	Males	Females	Males	Females	Males	Females	Males	Females	Males	Females
Number of animals	28	28	30	26	28	28	28	28	27	29	15	15	15	15	15	15	15	15
Numbers of litters	27	28	29	26	28	28	28	28	26	28								
Number of pup/litters	6	6	6	6	6	6	6	6	6	6								
Number of animals tested up to PND25	21	21	23	19	21	21	21	21	20	22								
Numbers of litters tested up to PND25	21	21	22	19	21	21	21	21	19	21								
Number of animals tested at PN73	7	7	9	6	7	7	7	7	6	8	5	5	5	5	5	5	5	5
Numbers of litters tested at PND73	7	7	9	6	7	7	7	7	6	7								
Number of animals tested at PND136	7	7	7	7	7	7	7	7	7	7								
Numbers of litters tested at PND136	7	7	7	7	7	7	7	7	7	7	5	5	5	5	5	5	5	5
Time of developmental testing	PND5-PND25																	
Time of behavioral testing	week before endpoint for PND73 and PND136										week before endpoint for D73 and D136							
Endpoint	PND11; PND26; PND73; PND136										D3; D73; D136							

**Table 2:** Effects of HCQ treatment on LC3 and SQSTM1/p62 proteins tissular level at several endpoints of juvenile experiment.

Organ			Tibial Anterior Muscle				Draining Lymph Nodes				Spleen				Liver				Brain			
			Group (mg HCQ/kg)				Group (mg HCQ/kg)				Group (mg HCQ/kg)				Group (mg HCQ/kg)				Group (mg HCQ/kg)			
Endpoint	Gender	Protein	15	30	50	70	15	30	50	70	15	30	50	70	15	30	50	70	15	30	50	70
**PND11**	**♂**	LC3-I	-	-	-	↓	-	-	-	-	-	-	-	-	-	-	-	-	-	-	↓	↓
		LC3-II	-	↓	↓	↓	-	-	-	-	-	-	-	-	-	-	-	-	↓	↓	↓	↓
		SQSTM1/p62	-	-	↓	-	-	-	-	-	-	↑	-	-	-	-	-	-	-	-	-	-
	**♀**	LC3-I	-	-	-	-	↑	-	-	-	-	-	-	-	-	-	-	-	-	-	-	-
		LC3-II	-	-	-	-	-	↑	-	-	-	-	-	-	-	-	-	-	-	-	-	-
		SQSTM1/p62	-	-	-	-	↑	↑	-	-	-	-	-	-	-	-	-	-	-	-	-	-
**PND26**	**♂**	LC3-I	-	-	-	-	-	-	-	-	↑	-	-	-	-	-	-	-	↑	-	↑	-
		LC3-II	-	↑	-	-	-	-	-	-	-	-	-	-	-	-	-	-	↑	↑	↑	-
		SQSTM1/p62	-	-	-	-	-	-	-	-	↑	-	-	↓	-	-	-	-	↑	-	↑	↑
	**♀**	LC3-I	-	-	-	-	↑	-	-	↓	-	-	-	-	-	-	-	-	-	-	↑	-
		LC3-II	-	-	-	-	-	-	-	-	-	-	-	-	-	-	-	-	-	-	↑	-
		SQSTM1/p62	↑	↑	-	-	-	-	-	-	-	↓	↓	↓	-	-	-	-	-	-	-	-
**PND73**	**♂**	LC3-I	-	-	-	-	-	-	-	-	-	-	-	-	-	-	-	-	-	-	-	-
		LC3-II	-	-	-	-	-	-	-	-	-	-	-	-	-	-	-	-	-	-	-	-
		SQSTM1/p62	-	-	-	-	-	-	-	-	-	-	-	-	-	-	-	-	-	-	-	-
	**♀**	LC3-I	-	-	-	-	-	-	-	-	-	-	-	-	↓	-	-	↓	-	-	-	-
		LC3-II	-	-	-	-	-	↑	-	-	↓	↓	-	-	-	-	-	-	-	-	-	-
		SQSTM1/p62	-	-	-	-	-	↑	-	↓	↓	↓	-	-	-	-	-	-	-	-	-	-
**PND136**	**♂**	LC3-I	-	-	-	-	↑	↑	↑	↑	-	-	-	-	-	-	-	-	-	-	-	-
		LC3-II	-	-	-	-	-	↑	-	↑	-	-	-	-	-	-	-	-	-	-	-	-
		SQSTM1/p62	-	-	-	-	-	-	-	-	-	-	-	-	-	-	-	-	-	-	-	-
	**♀**	LC3-I	-	↓	↓	-	-	-	-	-	-	-	-	-	↑	↑	-	↑	↑	-	-	↓
		LC3-II	-	-	↓	-	-	-	-	-	-	-	-	-	↑	↑	↑	↑	-	-	↓	↓
		SQSTM1/p62	-	-	↓	-	-	-	-	-	-	↑	-	-	-	-	-	-	-	-	-	-

Standardized protein level were analysed by Kruskal-Wallis test. Mann-Whitney was used for multiple comparisons. *n* = 5 mice/sex/group. -: no statistical difference compared to controls. ↑: statistical protein accumulation compared to controls (*p* < 0.05). ↓: statistical protein depletion compared to controls (*p* < 0.05).

**Table 3:** Effects of HCQ treatment on LC3 and SQSTM1/p62 proteins tissular level at several endpoints of the adult experiment.

Organ			Tibial Anterior Muscle			Draining Lymph Nodes			Spleen			Liver			Brain		
			Group (mg HCQ/kg)			Group (mg HCQ/kg)			Group (mg HCQ/kg)			Group (mg HCQ/kg)			Group (mg HCQ/kg)		
Endpoint	Gender	Protein	30	50	70	30	50	70	30	50	70	30	50	70	30	50	70
**D3**	**♂**	LC3-I	-	-	-	-	↑	↑	-	-	-	-	↓	↓	-	-	-
		LC3-II	-	-	-	-	-	-	-	-	-	-	-	↓	-	-	-
		SQSTM1/p62	-	-	-	-	-	-	-	-	-	-	↓	↓	-	-	-
	**♀**	LC3-I	-	-	-	-	-	↑	-	-	-	-	-	-	-	-	-
		LC3-II	-	-	-	-	↑	↑	-	-	-	-	-	-	-	-	-
		SQSTM1/p62	-	-	-	-	-	↑	-	-	-	-	-	-	↓	-	-
**D73**	**♂**	LC3-I	-	-	-	-	-	-	-	-	-	-	-	-	-	-	-
		LC3-II	-	-	-	-	-	-	-	-	-	-	-	-	-	-	-
		SQSTM1/p62	-	-	↓	-	-	-	-	↓	-	-	↓	↓	-	-	-
	**♀**	LC3-I	-	-	-	-	-	-	-	-	-	-	-	-	↓	-	-
		LC3-II	-	-	-	-	-	-	-	-	-	-	-	-	-	↓	-
		SQSTM1/p62	-	-	-	-	-	-	-	-	↓	-	-	-	-	-	-
**D136**	**♂**	LC3-I	-	-	-	-	↑	↑	-	-	-	-	-	↓	↓	-	↓
		LC3-II	-	-	-	↑	↑	-	-	↓	-	-	-	↓	↓	-	↓
		SQSTM1/p62	-	↓	-	↓	-	-	↓	↓	↓	-	-	↓	-	-	-
	**♀**	LC3-I	-	-	-	-	↑	-	-	-	-	-	↑	↑	-	-	-
		LC3-II	-	-	-	-	-	-	-	-	-	↑	↑	↑	-	-	-
		SQSTM1/p62	-	-	-	-	-	-	-	-	↓	↑	↑	↑	-	-	-

Standardized protein level were analysed by Kruskal-Wallis test. Mann-Whitney was used for multiple comparisons. *n* = 5 mice/sex/group. -: no statistical difference compared to controls. ↑: statistical protein acummulation compared to controls (*p* < 0.05). ↓: statistical protein depletion compared to controls (*p* < 0.05).

## Supplementary Materials

Supplementary materials can be found at https://www.mdpi.com/2227-9059/8/3/47/s1.

## Abbreviations

Al	Aluminium
ATG	Autophagy related
CQ	Chloroquine
HCQ	Hydroxychloroquine
ip	Intra-peritoneal
LC3	Microtubule-associated protein 1A/1B-light chain 3
MMF	Macrophagic myofasciitis
PND	Post-natal day
PVDF	Polyvinylidene difluoride
ROS	Reactive oxygen species
RT	Room temperature
TA	Tibialis anterior
